# Investigating a Non-Mesh Mosquito Net among Outdoor Sleeping Nomadic Communities in Kenya

**DOI:** 10.4269/ajtmh.14-0458

**Published:** 2015-11-04

**Authors:** Georgia R. Gore-Langton, James Mungai, Nfornuh Alenwi, Abdullahi Abagira, Owen M. Bicknell, Rebecca E. Harrison, Farah Amin Hassan, Stephen Munga, Katie Eves, Elizabeth Juma, Richard Allan

**Affiliations:** The MENTOR Initiative, Crawley, United Kingdom; The MENTOR Initiative, North Eastern Province, Kenya; Ministy of Health, Garissa, Kenya; The MENTOR Initiative, Maban, South Sudan; Centre for Global Health Research, Kenya Medical Research Insitute, Kisumu, Kenya; Division of Malaria Control, Ministry of Public Health, Nairobi, Kenya

## Abstract

Rising reports of exophagic malaria vectors make even more pressing the need for alternatives to traditional, mesh, long-lasting insecticidal nets (LLINs) designed for indoor sleeping and often inadequate in the protection of outdoor-sleeping populations. This study tests and evaluates the retention, utilization, and durability of novel, non-mesh nets designed for outdoor use. Longitudinal, cross-sectional surveys were conducted, the physical condition of nets was assessed, and bio-efficacy and insecticide content were tested. At 22 months, retention was 98.0%; 97.1% of nets fell within the World Health Organization (WHO) category of being in “good” condition; none were in the “torn” category. At 18 months post-distribution, 100% of nets had at least WHO Pesticide Evaluation Scheme (WHOPES)-acceptable levels of insecticide, this proportion was 66.7% at 22 months. This novel mosquito net has the potential to provide a durable and context-specific tool to prevent malaria among traditionally hard-to-protect and highly vulnerable populations.

## Introduction

Malaria is estimated to have caused 584,000 deaths in 2013; in the same year, there were an estimated 198 million cases of clinical malaria.^1^ Malaria control and treatment appears as one of the 2015 Millennium Development Goals as “Goal 6: Combat HIV/AIDS, malaria and other diseases.”^2^ An estimated $2.7 billion was spent globally on malaria control in 2013, just over half of the estimated amount needed to achieve universal coverage of malaria control ($5.1 billion).^1^

Long-lasting insecticidal nets (LLINs) have been shown to be a highly effective method of malaria prevention^3^–^7^; however, this effectiveness is often limited as the standard LLIN is not designed for use in all contexts. Physical and chemical durability in operational conditions vary greatly between location and population, and are often far from the physical and chemical durability seen in controlled settings.^8^–^11^ In the semiarid regions of Africa, which constitute a significant proportion of the world's malarious zones, live many nomadic, outdoor-sleeping populations. Nomadic populations, defined here as groups of people with no fixed home who move according to the seasons and in search of water, food, and pasture, have been estimated at 50–100 million persons globally^12^ with over 60% found in Africa, and make up approximately 19% of the population in Kenya.^12^–^14^ The characteristic mobility of these populations is often associated with sleeping outdoors, an environment unprotected by indoor residual spraying (IRS) and in which LLINs designed for indoor sleeping are unsuitable or inadequate. Outdoor use and ultraviolet (UV) exposure increase the rate and degree of insecticide degradation,^15^ hence the World Health Organization (WHO) recommendation to dry the nets in the shade. These harsh climates mean that nomads are rapidly left with inefficient nets offering little, if any, protection from the bites of malaria vectors.

Increasing reports of exophagic biting of vectors have been associated with large-scale, blanket distribution of LLINs designed for indoor use.^16^–^20^ The exact reasons for these changes vary and are subject of uncertainty and controversy. Although questions still remain, it is clear that increases in outdoor biting, and thus outdoor transmission, are of great concern, especially to nomadic populations that often sleep outside and typically have reduced access to health services, exposing them to greater risks of both malaria morbidity and mortality.

Nomadic, outdoor sleepers and exophagic biting of malaria vectors are two challenges to traditional LLINs and their effectiveness, especially as they overlap geographically. It is, therefore, necessary to understand what role novel interventions can play in providing protection from the bites of malaria vectors in these contexts.^16^,^21^,^22^

Garissa County in northeastern Kenya, the setting of this study, is an area of very harsh, semiarid terrain. This highly insecure area, and the large number of nomadic communities that call it home, displays many factors that in similar settings have highlighted the weaknesses of standard LLINs.^8^,^10^,^23^ Greater understanding of the effects of outdoor sleeping and the nomadic lifestyle on the physical integrity and insecticidal activity of nets would allow control programs serving these individuals to more accurately plan and budget for distribution and redistribution campaigns in similar contexts and would inform manufacturers on how to improve net design.

**Figure 1.**
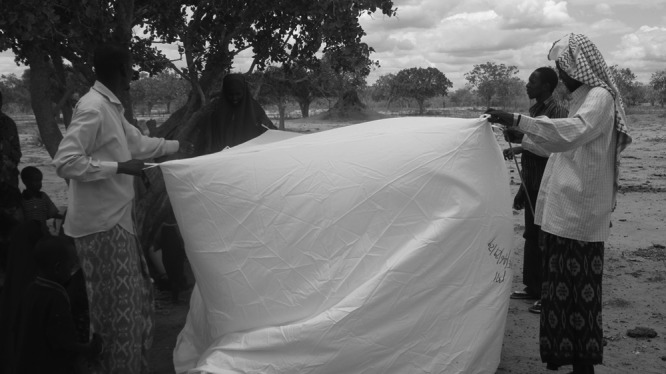
A Dumuria net being displayed in the field.

PermaNet^®^ Dumuria (hereafter referred to as a Dumuria net) is a mosquito net produced by Vestergaard, Lausanne, Switzerland, intended for use both indoors and outdoors. This net is based on the PermaNet^®^ 2.0, which is fully evaluated and recommended by the WHO Pesticide Evaluation Scheme (WHOPES)^24^; the only differences being unlike a typical 156-mesh LLIN, this net is made of a non-mesh, opaque, bed sheet-like fabric (Figure 1

) and added to the insecticide in the Dumuria net (not found in the PermaNet 2.0) are UV protectants designed to make the insecticide more resilient to sunlight exposure. This net has previously been distributed to the nomadic population of South Sudan, where extremely high levels of acceptability were found when compared with standard LLINs (these results have not been published in peer-reviewed journals) (P. Guillet, unpublished data).

We present here results from a 22-month longitudinal study into the utilization and durability of Dumuria nets among nomadic communities in Garissa County, northeastern Kenya. WHOPES guidelines on investigating LLIN durability were followed as far as possible; the only difference being follow-up until 22 months rather than the 36 months required to qualify a net as an LLIN. Standardized outcome measures were chosen.

## Methods

### Study area and population.

**Figure 2.**
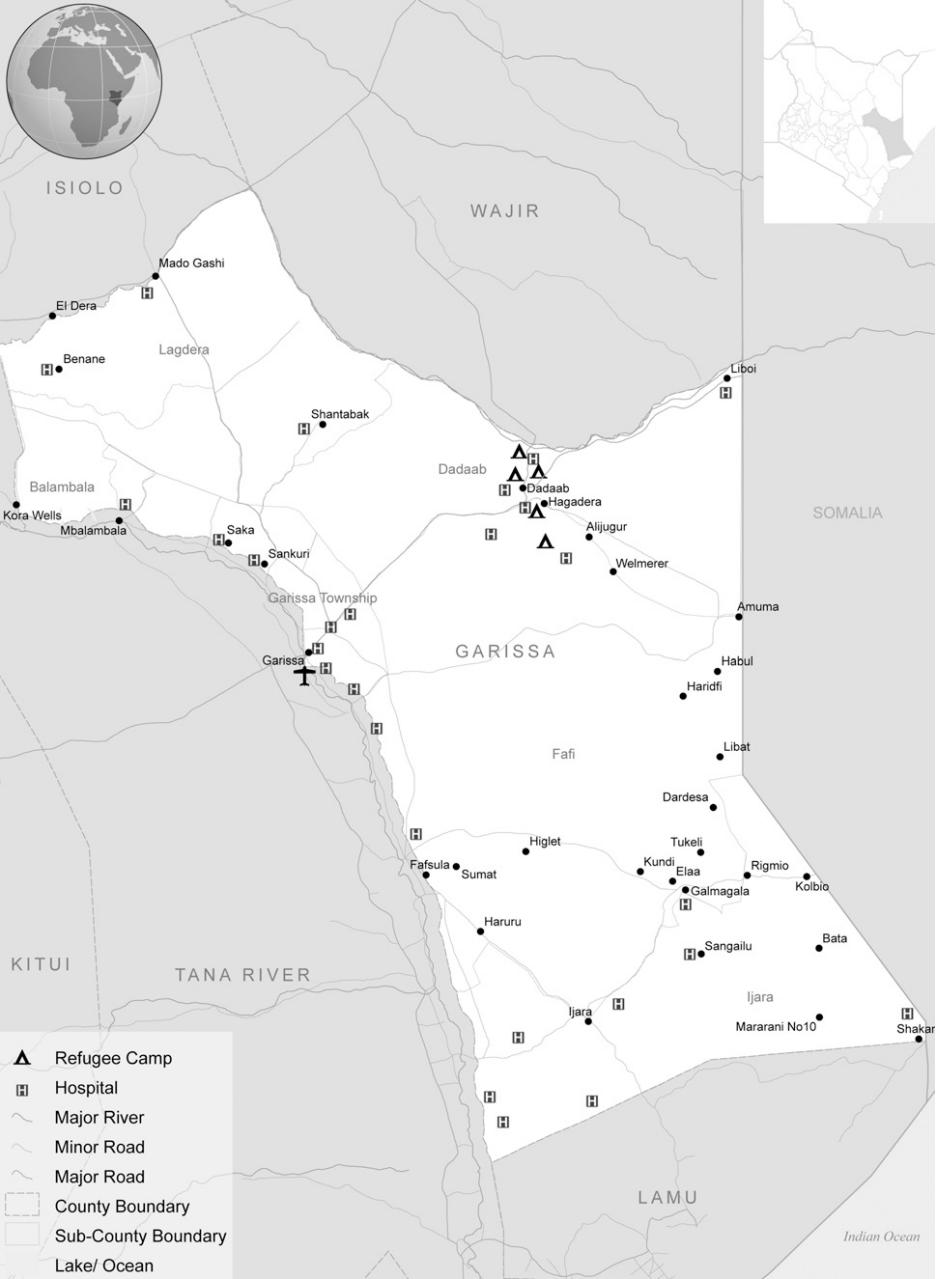
Map of the study region.

Garissa County is situated in North Eastern Province, Kenya; it is split into three administrative districts, Garissa, Lagdera, and Fafi, with a total of 11 divisions (Figure 2

). The climate is semiarid with a range in temperature from 21°C to 39°C in 2012^25^ and an annual average bimodal rainfall (rainy seasons from March to May and from September to October) of 250–300 mm.

The population according to the last national census in 2009 was 623,060.^26^ The population was estimated to have grown to 715,312 in 2014 based on the United Nations Children's Fund (UNICEF) prediction of a 2.8% annual growth rate.^27^ Outside of cities and large towns, 60% of the population are nomadic,^12^–^14^ defined here as people living within a temporary shelter typically made of a stick frame and cattle skins, dismantled after several months in one place and moved on as their cattle are moved to new grazing land. Shelters are often used during the day to provide shade and protection from the sunshine. At night, depending on temperature and rainfall, individuals may choose to sleep either under shelters or outside (based on observations made during study implementation). The use of the word “household” in this study refers to the area where nomadic families are based and includes whole family units, often consisting of several shelters.

Malaria transmission is seasonal and epidemic prone.^28^ The primary vectors in the region are mainly exophagic and exophilic, such as *Anopheles coustani* and *Anopheles arabiensis*.^20^,^29^–^31^ Both *Plasmodium falciparum* and *Plasmodium vivax* are present with *P*. *falciparum* accounting for 98% of infections.^32^ In 2013, there were 4,519 clinical cases of malaria and 1,321 confirmed cases of malaria in Garissa, this is a 92% reduction in both clinical and confirmed cases since 2010 (Kenyan Ministry of Health, unpublished data).

### Net distribution.

In September 2011, a total of 13,922 Dumuria nets were distributed to 8,511 nomadic households. This provided an average coverage rate of 1.64 nets per household. All recipients received malaria prevention education to encourage correct usage and prioritization of use by those most vulnerable to malaria, that is, those under the age of 5 years and pregnant women. Each net was assigned a unique identifier and added to a master census list along with a household code and global positioning satellite coordinates.

### Consent.

This study was planned with and approved by the Department of Malaria Control, Ministry of Health in Garissa, and the Kenyan Medical Research Institute and the Ethic Review Committee. All nomadic settlements involved in the survey were informed in advance of the distribution and the surveys. Approval was obtained from local chiefs and traditional authorities and informed consent was obtained from the head of each household surveyed.

### Study design and sample size.

From September 2011 to July 2013, a prospective longitudinal study was conducted with cross-sectional household surveys to assess Dumuria net utilization and physical integrity at months 6, 12, 18, and 22, and net retention at months 18 and 22. Nets were randomly selected from the total 13,922 distributed Dumuria nets using a two-stage cluster sampling method with villages or household clusters as the primary sampling unit and individual households as the secondary sampling unit.

Sample size was calculated using the formula [Np(1 − *p*)]/[(d^2^/Z^2^_1-α/2_ × (N − 1) + *p* × (1 − *p*)]. The sample of Dumuria nets required is based on the conservative estimate of the outcome of retention and usage of 60% of Dumuria (as supported by the literature^33^–^35^) (*P* = 0.06) and a 95% confidence interval (CI). Thirty clusters were randomly selected using the probability proportional to size method. Simple random sampling was used to select 10 Dumuria nets from each cluster. A total of 300 Dumuria nets were sampled each time at 6, 12, 18, and 22 months up to a total of 1,200 nets sampled. The unique identification number of each net was cross-referenced to obtain the household number. Destructive sampling was followed with the Dumuria net being removed from the master list after being surveyed.

Insecticidal activity was assessed at each survey time point by randomly selecting 30 Dumuria nets, as per the WHOPES guidelines, the nets were sent for testing by WHO cone bio-efficacy and insecticidal content testing at an external laboratory. Each net given up for testing was replaced with a new Dumuria net not included on the master list of nets for future surveys.

### Field procedures.

The households of the selected nets were visited, the surveys were administered, and the nets were inspected. The size, quantity, and position of holes were recorded. Size was assessed by the simple thumb, fist, or head method that places holes into four categories: smaller than thumb (0.5–2 cm), larger than a thumb but smaller than a fist (2–10 cm), larger than a fist but smaller than a head (10–25 cm), and larger than a head (> 25 cm).These categories are then weighted 1, 23, 196, and 578 from less than a thumb to larger than a head, respectively. The number of holes in each category multiplied by the weight of the category gives a proportional hole index (pHI). This index is an attempt to standardize research on LLINs and is developed from previous work^8^,^33^,^36^ and can be found in the WHO guidelines for LLIN durability testing in the field.^37^

The bio-efficacy and insecticide content of net samples were assessed by Vestergaard Vector Control Laboratories (253/9 Minh Khai, Hai Ba Trung, Hanoi, Vietnam and Vestergaard-Frandsen NMIMR Vector Testing Center, Noguchi Memorial Institute for Medical Research, University of Ghana, Legon, Accra Ghana). Samples of 30 randomly selected nets at each survey period were sent to be tested. All samples were blinded so the laboratory did not know the age or any other details of the net sample. Once results were received they were unblinded and decoded, this method reduced the risk of bias at the laboratory. Bio-efficacy in terms of knock down (KD-60) and total mortality were tested using a standard cone test according to WHO guidelines.^38^ Individual, unfed, 2-day-old female mosquitoes (of a strain susceptible to pyrethroids) were allowed contact with samples in WHO standard cones. After a defined exposure time of 3 minutes, mosquitoes were removed and held in plastic cups at a temperature of 25°C ± 2°C and a relative humidity of 75% ± 10% and given access to honey solution of 10%. Knock down rate was observed at 60 minutes and mortality was observed at 24 hours after exposure. A negative control (untreated sample) was run concurrently; for control samples, where knock down and/or mortality exceeded 20%, all results were discarded and where knock down and/or mortality was between 5% and 20%, Abbott's formula was applied to adjust for mortality not associated with insecticide treatment. The test method complied with the WHO guidelines for laboratory and field testing of long-lasting insecticidal mosquito nets.^38^

Insecticide content was assessed by quantifying the amount of active ingredient per gram of Dumuria net. Samples were extracted by refluxing with xylene. The solvent was evaporated and the residue was dissolved in mobile phase using a mixture of n-hexane and 1,4-dioxane (93:7, v/v). Deltamethrin content was determined by normal-phase high-performance liquid chromatography on a silica column using dibutyl phthalate as an internal standard with the UV detection at 236 nm. The procedure is a validated and ISO IEC 17025-certified test. The test method complies with method CIPAC 333/LN.

### Data handling and statistical analysis.

Quantitative data were double entered into Epi-Info (WHO/CDC, 2000) and analyzed in SPSS (SPSS Inc. Released 2007. SPSS for Windows, Version 16.0; SPSS Inc., Chicago, IL) and STATA (StataCorp. 2013. Stata Statistical Software; Release 13. StataCorp LP, College Station, TX). Data were summarized using proportions and means and medians, where appropriate. Comparisons of proportions between categorical variables were performed by χ^2^ test, using Fisher's exact test for significance where appropriate. Comparisons between quantitative variables were performed by simple regression. Mean rank scores, which displayed non-normal distributions, were compared by the nonparametric Kruskal–Wallis test with post hoc Mann–Whitney tests performed where appropriate and the Bonferroni correction (dividing the *P* value to be achieved for significance by the number of paired comparisons made) was applied. Binary logistic regression was used to test for significance between potential explanatory variables and the dichotomous outcome variables of the existence of holes. Significance tests were determined at the 5% level and 95% CI were calculated throughout.

Retention and attrition rates, developed by the WHO,^37^ were calculated at month 18 and 22 survey time points. Retention was calculated by dividing the number of households with the Dumuria net present and available for sleeping under by the total number of sampled households to which Dumuria nets were distributed and multiplying by 100. Three attrition rates were calculated according to the following formulas:

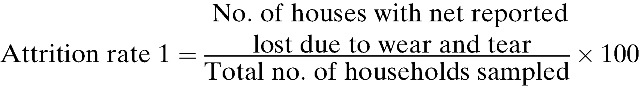


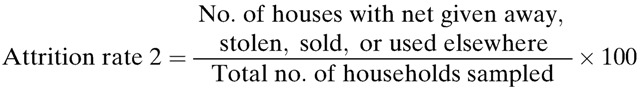


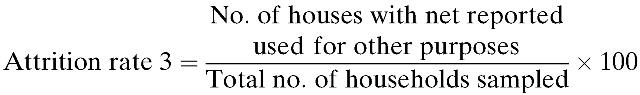


All analytical procedures took into account the two-stage cluster sampling design by using the svy family of commands in STATA and setting the primary sampling unit as the clusters.

## Results

### Household characteristics.

Overall, a total of 1,197 Dumuria nets were sampled from as many households, with a total of 7,365 inhabitants. The mean number of persons per household was 6.14, and 31.8% (95% CI: 30.7, 32.8) of the total population were under 5 years old. The median number of sleeping places per household was 2 (range: 2–22). Fifty-four point three percent (95% CI: 49.4, 59.7) of respondents had attended at least primary level education, 96.4% (95% CI: 93.3, 98.1) practiced open defecation, and 92.3% (95% CI: 90.6, 93.6) used an unimproved water source.

### Retention and attrition rates.

At 22 months, the retention rate was 98.0% (95% CI: 96.4, 99.5), attrition rate 1 was 0.3% (95% CI: 0.3, 0.9), attrition rate 2 was 1.3% (95% CI: 0.02, 2.6), and attrition rate 3 was 0.3% (95% CI: 0.3, 0.9).

### Net utilization.

A total of 98.4% (95% CI: 97.3, 99.0) of respondents reported using the Dumuria net for sleeping with 60.8% (95% CI: 56.4, 65.0) using the net every night and 0.4% (95% CI: 0.2, 1.2) reporting not using the net at all. About 97.4% (95% CI: 96.0, 98.4) of respondents reported sleeping under the net the previous night. Five reasons were listed for not sleeping under the net the previous night: too hot, no malaria, no mosquitoes, net not available, and used another net.

The majority of respondents, 74.1% (95% CI: 70.2, 77.6), reported sleeping under the net all year, 25.2% (95% CI: 21.8, 28.9) only during the rainy season, and 0.8% (95% CI: 0.3, 1.7) only during the dry season. When asked whether the net was used away from the household, 58.1% (95% CI: 52.2, 63.7) reported only using the net in the household, 20.5% (95% CI: 17.3, 24.1) took the net into the fields, 15.6% (95% CI: 12.2, 19.9) took the net into the forest, and 2% (95% CI: 1.2, 3.4) took the net to a farm hut. Among those that reported using the net away from the household, 21.2% (95% CI: 17.2, 26.0) did so only during the rainy season, 0.9% (95% CI: 0.3, 2.3) only during the dry season, and 77.9% (95% CI: 73.1, 82.0) reported sleeping away from the household all year-round.

A majority of the respondents, 86.5% (95% CI: 83.1, 89.3), reported tucking the net under the sleeping mat during use and 52.8% (95% CI: 47.6, 57.8) reported washing their nets (with 78 [6.5%] values missing). Sixty-five percent (95% CI: 61.2, 68.9) of those that reported washing their nets used a local bar soap and 12.9% (95% CI: 10.4, 15.8) used no soap at all. Among those respondents who washed their nets, 19.1% (95% CI: 14.4, 24.9) scrubbed their nets and 45.9% (95% CI: 40.7, 51.3) dried their nets in the sun compared with 53.1% (95% CI: 47.5, 58.5) who reported drying their nets outside in the shade.

### Physical condition of Dumuria nets.

**Figure 3.**
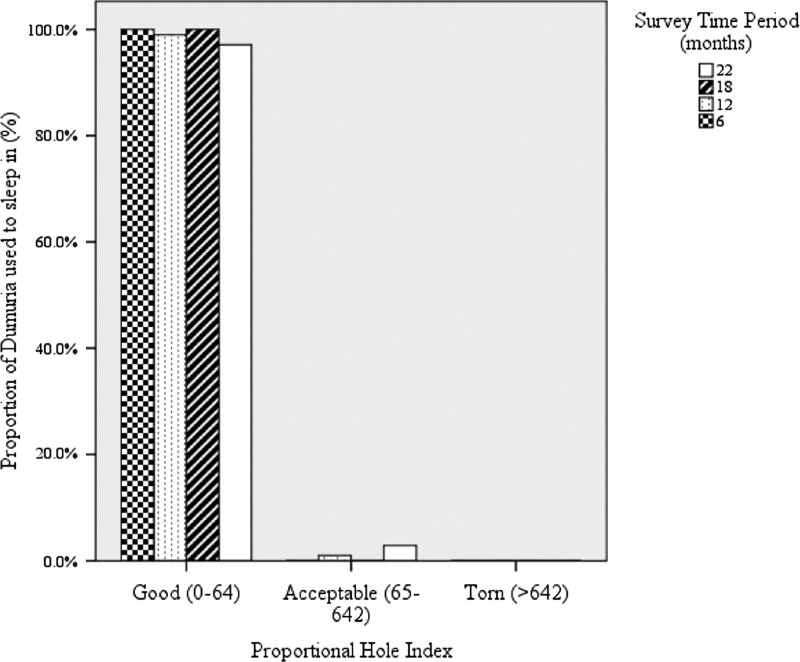
The performance profile of Dumuria nets used for sleeping under them represented by categorical proportion hole index (pHI) and according to survey time points.

At the end of the study period (month 22), 15.5% (95% CI: 10.3, 22.6) of the nets used to sleep under and available for examination had at least one hole. The median pHI was 0 with an interquartile range of 0–0 and a range from minimum 0 to maximum 196. In the WHO-defined “good” range of 0–64, pHI were 97.1% (95% CI: 94.0, 98.6) of nets, 2.9% (95% CI: 1.4, 6.0) were in the “acceptable” range of 65–642 pHI, and 0% were in the “torn” range of > 642 pHI (Figure 3

).^37^

Table 1 shows that after 22 months of use, horizontal holes were the most frequent type of holes and the largest proportion of holes were located in the lower section of the nets. Of the four categories of hole sizes, size 1 (0.5–2 cm) was the most frequently found size, there was only one hole of category 4 size (> 25 cm) and this was found in a net surveyed at 12 months. Category 1 and 2 holes were mostly located in the lower sections of the net whereas category 3 holes tended to be found in equal numbers across all sections of the net.

Logistic regression, adjusted for the village clusters in the study design, was conducted to assess known possible predictors of the development of holes. The binary variable of either having holes or not, established by net inspection, was set as the dependent variable and various independent variables were added to the model. The final model revealed four significant predictors of which taking the net to a farm hut and using an open flame for heating, cooking, or lighting were associated with the greatest increase in odds of a net having at least one hole (Table 2).

The results of the nonparametric Kruskal–Wallis test and, when necessary, the post hoc Mann–Whitney test showed the following as having significantly different mean pHI ranks: taking the net to the forest 378.97 compared with not using the net away from the household 358.20, *U* = 4.058 E4, *P* = 0.018; using an open flame 563.51 and not using an open flame 472.85, *U* = 6.423 E4, *P* < 0.001; and nets that were found outside 510.83 and nets that were found inside 489.38, *U* = 8.488 E4, *P* = 0.026.

### Insecticide content and insecticidal activity.

After 22 months of use, the mean deltamethrin content (mg/m^2^) for the Dumuria samples was 35.6 (95% CI: 21.9, 49.3), a fall of 64.6 from the baseline mean of 100.2, and the range was 93 (minimum 3, maximum 96) (Table 3). The bio-efficacy tests on the same samples at month 22 resulted in a mean KD-60 (%) of 79.8 (95% CI: 67.6, 92.0), and a mean total mortality (%) of 60.0 (95% CI: 46.8, 73.3). The proportion of samples passing either WHO cutoff of KD-60 ≥ 9 5% or total mortality ≥ 80%, and therefore being considered effective according to the WHOPES guidelines, was 100% at 6 months, 96.7% (95% CI: 82.8, 99.9) at 12 months, 100% at 18 months, and 66.7% (47.2, 82.7) at 22 months. Untreated “control” nets had KD-60 and total mortality measurements of 0% at all survey time points.

The results of Kruskal–Wallis tests, and post hoc Mann–Whitney tests in the case of a statistically significant difference in mean ranks, indicate that there is a significant difference in total mortality mean ranks between those using the net away from the household only during the dry season 28.25 and using the net away from the household only during the wet season 16.38, *U* = 52.5, *P* = 0.002.There is also a significant difference in mean ranks between those who used an open flame for cooking, heating, or lighting where the net was found and those who did not use an open flame: deltamethrin at 41.29 compared with 59.30, *U* = 722, *P* = 0.011; and mortality at 34.73 compared with 61.35, *U* = 552, *P* < 0.001.

## Discussion

After almost 2 years of varied field conditions and high reported utilization and acceptance rates,^39^ the retention rate of Dumuria nets is extremely high and the attrition rates are very low, indicative of a durable and fit-for-purpose net for outdoor sleeping. When nets were found damaged, time and factors associated with net use have been shown to be predictors of such damage.

Comparisons with similar studies show that Dumuria nets are much more durable than equivalent nets made of mesh. The survival rate of 98% at 22 months and very low attrition rates are far more impressive than results from other studies; a study in Ethiopia reported approximately 31% of LLINs discarded after 3 years (a third of which were discarded within a year).^33^ Another study in eastern Chad found that after 14 months of use in semiarid conditions less than one-third (30.5%) of the nets were in a serviceable condition,^8^ whereas a study testing the physical durability of PermaNet 2.0 LLIN, on which the Dumuria net is based, in Ethiopia found that at 3–6 months 54.5% of the LLINs had at least one hole, at 26–32 months this proportion increased to 92.5%.^10^ Assessment of the durability of Olyset^®^ nets in Benin found similarly low retention and attrition rates when compared with the findings of this study; retention was 57% at 18 months post-distribution, and at the same time the proportion of nets with any hole in varied by region between 72% and 93%.^40^

The chemical composition of the Dumuria nets also proved to be resilient to high levels of utilization, washing, scrubbing, and exposure to sunshine; 18 months after distribution, 100% of nets passed WHO insecticide acceptability thresholds, this proportion only decreased at 22 months of use to 66.7%. Although insecticide has been shown to be better on standard nets used indoors,^36^,^41^ no nets have previously been shown to be as durable when used outside, in as harsh conditions, and for as long a period as the Dumuria nets.^8^,^10^,^33^,^40^

Potential limitations of this study arise because of its prospective nature. The Hawthorne effect of study beneficiaries being more inclined to retain their nets than dispose them, as they know they are the subject of research , may have had an effect on the study results.^42^ It is also possible that grateful beneficiaries would report a net that was discarded because of damage as given away to friends/neighbors so as not to offend the researchers, thus diminishing attrition rate 1 and inflating attrition rate 2.^42^ Had a common factor linked nonrespondents to either a lack of nets or damaged nets, a lack of recording and follow-up of these nonrespondents could have distorted results.^43^

The standardized nature of the study in accordance with WHO protocols allows for comparison of results with similar products. The majority of holes being found in the lower region of the nets is consistent with the findings of previous studies^10^,^36^ and is likely due to the practice of tucking the net under the sleeping mat at night. The relatively frequent occurrence of burn holes and the use of open flames being a significant predictor of poor chemical performance highlight the importance of further research into this possible interaction. If these results continue to be replicated, they could help to inform information, education, and communication and behavior change communication campaigns regarding net use, in general and more specifically, in relation to the use of open flames, and prompt manufacturers to develop future net designs so as to protect against such damage.

Another limitation of the study is the absence of the measurement of insecticidal efficacy of the nets against wild mosquitoes collected in the study area. The testing of the nets in a laboratory using female mosquitoes known to be susceptible to pyrethroids goes a certain extent in analyzing the insecticidal efficacy of the nets over time, but it cannot be assumed to give a completely accurate measurement of the insecticidal efficacy of the nets in the field and against the wild-type mosquitoes found in the field. While there are to date no reports of pyrethroid-resistant mosquitoes in northeastern Kenya,^44^ extrapolating from data collected in other areas of the country,^19^,^45^,^46^ the likelihood of at least some members of the mosquito population in the study area being resistant to pyrethroids is high. This study would benefit from future follow-on studies into the insecticidal efficacy of the Dumuria nets over time against wild-type mosquitoes collected from the study area.

In a time of strained and even diminishing budgets driving down the price of LLINs may appear a quick-fix solution. However, the relationship between reduced LLIN cost and LLIN quality and durability may render this approach counterproductive as more frequent redistribution campaigns are required at significant cost. It may also be reckless as, if not well planned, gaps between redistribution campaigns leave people unprotected from malaria infection, morbidity, and mortality. The distribution of traditional LLINs designed for indoor use to nomadic populations who use them outdoors may also have implications on the ever pressing issue of mosquito resistance to insecticides. As these nets quickly lose their insecticidal activity in the sunshine, mosquitoes are exposed to nets containing sublethal doses of insecticide; this could pose a significant factor in the development of insecticide resistance,^47^,^48^ but will require studies to provide evidence as such.

In other contexts, particularly in southeast Asia, long-lasting insecticidal hammocks have been explored^50^,^51^; however, so far there have been no other LLINs designed for use in the semiarid conditions of the Sahel. The seemingly high cost of Dumuria nets (US$13) should be regarded as an investment in the long-term protection of vulnerable people living in some of the harshest climates on earth where no other options have so far proven to be as effective or as durable. This study not only highlights Dumuria nets as the best option currently available for outdoor sleepers and harsh environments, but also highlights the importance of context-specific LLINs, which take in to consideration the very nuanced needs and preferences of the communities they are serving, thus providing an argument against the traditional large-scale, blanket net distribution campaigns.

## Figures and Tables

**Table 1:** The frequency and proportion of types and positions of holes among the total number of nets with holes (*N* = 43) at 22 months

	Number of nets	Proportion (%) of total nets with holes (*N* = 43)	95% CI
Type of hole			
Horizontal hole	18	41.9	27.0, 57.4
Burn hole	13	30.2	17.5, 46.9
Hole at hanging	9	20.9	13.4, 31.1
Open seams	8	18.6	9.4, 33.4
Whole section missing	4	9.3	3.7, 21.4
Rodents	1	2.3	0.3, 17.6
Category 1 holes (0.5–2 cm)			
Roof	5	11.6	3.9, 25.1
Upper	3	7.0	1.5, 19.1
Lower	9	20.9	10.0, 36.0
Seams	2	4.7	0.5, 15.8
Category 2 holes (2–10 cm)			
Roof	1	2.3	0.05, 12.3
Upper	0	–	–
Lower	11	25.6	13.5, 41.2
Seams	2	4.6	0.5, 15.8
Category 3 holes (10–25 cm)			
Roof	2	4.6	0.5, 15.8
Upper	3	7.0	1.5, 15.8
Lower	1	2.3	0.05, 12.3
Seams	2	4.6	0.5, 15.8

CI = confidence interval.

**Table 2:** Multivariate logistic regression model of variables significantly associated (*P* < 0.05) with the presence of at least one hole in the net

Variable	OR (95% CI)	*P*
Months	1.07 (1.00, 1.1)	0.042
Net used away from home		
Not used away	Baseline	–
Taken to farm hut	13.7 (3.1, 61.1)	0.001
Taken to forest	3.3 (1.4, 8.0)	0.008
Where net found at time of survey		
Found inside	Baseline	–
Found outside	2.3 (1.5, 3.6)	0.001
Use of an open flame		
Open flame not used	Baseline	–
Open flame used	6.30 (3.0, 13.1)	0.001

CI = confidence interval; OR = odds ratio.

**Table 3:** DM, KD-60, and total mortality of sampled nets at each survey time point: 6, 12, 18, and 22 months

Test	Measure	Baseline	6 months (*N* = 29)	12 months (*N* = 30)	18 months (*N* = 30)	22 months (*N* = 30)
DM (mg/m^2^)	Mean (95% CI)	100.2	71.3 (62.8, 79.8)	40.3 (28.2, 52.4)	43.5 (32.6, 54.3)	35.6 (21.9, 49.3)
	Standard deviation	–	22.4	32.4	29.0	33.2
	Median (IQR)	–	68.0 (59.3, 80.0)	36.9 (14.7, 54.8)	49.3 (18.0, 66.0)	23 (6.0, 63)
KD-60 (%)	Mean (95% CI)	–	100	98.4 (96.7, 100)	100	79.8 (67.6, 92.0)
	Standard deviation	–	–	4.7	–	32.7
	Median (IQR)	–	100 (100, 100)	100 (100, 100)	100 (100, 100)	100 (64, 100)
Total mortality (%)	Mean (95% CI)	–	96.5 (94.8, 98.3)	98 (95.4, 100)	95.9 (91.6, 100)	60.0 (46.8, 73.3)
	Standard deviation	–	4.6	7.1	11.5	35.5
	Median (IQR)	–	98.0 (94.0, 100)	100 (100, 100)	100 (97, 100)	24 (73.5, 93.0)
Passed either WHO cutoff (%) (KD-60 ≥ 95% or total mortality ≥ 80%)			100	96.7 (82.8, 99.9)	100	66.7 (47.2, 82.7)

CI = confidence interval; DM = deltamethrin content; IQR = interquartile range; KD = knock down; WHO = World Health Organization.
